# Human Pentraxin 3 (PTX3) as a Novel Biomarker for the Diagnosis of Pulmonary Arterial Hypertension

**DOI:** 10.1371/journal.pone.0045834

**Published:** 2012-09-21

**Authors:** Yuichi Tamura, Tomohiko Ono, Masataka Kuwana, Kenji Inoue, Makoto Takei, Tsunehisa Yamamoto, Takashi Kawakami, Jun Fujita, Masaharu Kataoka, Kensuke Kimura, Motoaki Sano, Hiroyuki Daida, Toru Satoh, Keiichi Fukuda

**Affiliations:** 1 Department of Cardiology, Keio University School of Medicine, Tokyo, Japan; 2 Department of Rheumatology, Keio University School of Medicine, Tokyo, Japan; 3 Department of Cardiology, Juntendo University Nerima Hospital, Tokyo, Japan; 4 Department of Cardiology, Kyorin University School of Medicine, Tokyo, Japan; 5 Department of Cardiology, Juntendo University School of Medicine, Tokyo, Japan; University of Leuven, Rega Institute, Belgium

## Abstract

**Background:**

Although inflammation is an important feature of pulmonary arterial hypertension (PAH), the usefulness of local inflammatory markers as biomarkers for PAH is unknown. In this study, we tested whether plasma concentrations of human pentraxin 3 (PTX3), a local inflammatory marker, would be a useful biomarker for detecting PAH.

**Methods:**

Plasma PTX3 concentrations were evaluated in 50 PAH patients (27 with idiopathic PAH, 17 with PAH associated with connective tissue disease (CTD-PAH), and six with congenital heart disease), 100 age and sex-matched healthy controls, and 34 disease-matched CTD patients without PAH. Plasma concentrations of B-type natriuretic peptide (BNP) and C-reactive protein (CRP) were also determined.

**Results:**

Mean PTX3 levels were significantly higher in all PAH patients than in the healthy controls (4.40±0.37 vs. 1.94±0.09 ng/mL, respectively; *P*<0.001). Using a threshold level of 2.84 ng/mL, PTX3 yielded a sensitivity of 74.0% and a specificity of 84.0% for the detection of PAH. In CTD-PAH patients, mean PTX3 concentrations were significantly higher than in CTD patients without PAH (5.02±0.69 vs. 2.40±0.14 ng/mL, respectively; *P*<0.001). There was no significant correlation between plasma levels of PTX3 and BNP or CRP. Receiver operating characteristic (ROC) curves for screening PAH in patients with CTD revealed that PTX3 (area under the ROC curve 0.866) is superior to BNP. Using a PTX3 threshold of 2.85 ng/mL maximized true-positive and false-negative results (sensitivity 94.1%, specificity 73.5%).

**Conclusion:**

Plasma concentrations of PTX3 may be a better biomarker of PAH than BNP, especially in patients with CTD.

## Introduction

Despite the development of drugs that can bring about improvements in hemodynamics, exercise capacity, and quality of life, pulmonary arterial hypertension (PAH) remains a life-threatening disease with a poor prognosis. Recent guidelines [1], [2] encourage the use of screening examinations, such as an echocardiogram (UCG), in high-risk populations for the early detection of PAH [3].

To detect PAH in patients with connective tissue disease (CTD), the obvious screening tests are an UCG [4] and spirometry, including assessment of the diffusing capacity of the lung for carbon monoxide (DLCO) [5], [6]. Previous studies have suggested that B-type natriuretic peptide (BNP) and its N-terminal prohormone (NT-proBNP) are potential biomarkers for PAH [7], [8]. However, neither BNP nor NT-pro BNP are specific biomarkers of the degeneration of the pulmonary artery; rather, they are biomarkers of cardiac burden resulting from right heart failure.

In the present study, we demonstrate that human pentraxin 3 (PTX3) is a specific biomarker for PAH, reflecting pulmonary vascular degeneration, especially in patients with CTD. This is the first study in which the usefulness of PTX3 as a biomarker for PAH has been demonstrated.

Pentraxins are a family of evolutionarily conserved proteins [9]. They are divided into short and long pentraxins on the basis of their primary structure. C-Reactive protein (CRP) and serum amyloid P are the classic short pentraxins that are produced in the liver in response to systemic inflammatory cytokines. In contrast, PTX3 is one of the long pentraxins. It is synthesized by local vascular cells, such as smooth muscle cells, endothelial cells and fibroblasts, as well as innate immunity cells at sites of inflammation [10]. PTX3 plays a key role in the regulation of cell proliferation and angiogenesis [11]. In the field of cardiovascular diseases, increased serum PTX3 levels have been reported in patients with acute coronary syndromes. For example, increased plasma PTX3 levels have been reported in patients with acute myocardial injury in the 24 h after admission to hospital, with levels returning to normal after 3 days [12]. Similarly, PTX3 levels are higher in patients with unstable angina pectoris [13], with the changes in PTX3 levels found to be independent of other coronary risk factors, such as obesity and diabetes mellitus [13]. Finally, high serum PTX3 levels have been reported in patents with vasculitis, such as small-vessel vasculitis [14] and Takayasu aortitis [15], [16]. Thus, on the basis of these observations, we chose to investigate PTX3 as a potential biomarker for PAH.

## Methods

### Study Population

This study was approved by local ethical committee in Keio University Hospital (KEIO UNIVERSITY SCHOOL OF MEDICINE AN ETHICAL COMMITTEE, Tokyo, Japan), and all patients and controls who were enrolled in the study provided written informed consent. All patients with PAH and CTD in the present study were cared for at Keio University Hospital (Tokyo, Japan). Fifty consecutive PAH patients (27 with idiopathic or heritable PAH, 17 with CTD-associated PAH (CTD-PAH), and six with congenital heart disease-associated PAH) attending Keio University Hospital between January 2011 and July 2011 were eligible for inclusion in the study. As suggested by the Dana Point Classification system, diagnoses of PAH were made by performing right heart catheterization. The diagnostic criteria for PAH were based on the American College of Cardiology (ACC)/American Heart Association (AHA) guidelines [1]. Extensive diagnostic evaluations were also performed to exclude other types of pulmonary hypertension (Class 2–5 of the Dana Point Classification system 2008). Two different populations were recruited to the study as control groups. The first comprised 100 healthy blood donors from a published cohort [17], matched for sex and age (within 10 years) with the PAH patients. There were twice as many donors as PAH patients, and the donors served as the control group for PTX3 measurements. The second control group consisted of 34 disease matched CTD patients without PAH (ruled out on the basis of UCG or right heart catheterization results). CTD patients with or without PAH were classified as having either scleroderma (SSc) or non-SSc CTD and disease matched. The plasma analysis performed in the present study was approved by the institutional review board of Keio University Hospital.

### Assays

Serum markers (PTX3, CRP, BNP) were evaluated in all PAH patients during a single visit. Plasma concentrations of PTX3 were determined using a well-established, commercially available, highly sensitive and specific plasma ELISA using monoclonal antibodies (Perseus Proteomic, Tokyo, Japan) [13]. Plasma PTX3 concentrations were determined in healthy subjects using the same assay. No cross-reactions were observed with other pentraxins, including CRP. There were no missing data for PTX3, CRP, or BNP.

### Patient Assessments

Disease duration (months) in the present study was calculated from the time of the initial diagnosis of PAH. Hemodynamic parameters were also evaluated by right heart catheterization within 1 month of the collection of blood samples. Furthermore, mean pulmonary arterial pressure (mPAP) and pulmonary vascular resistance (PVR) were determined in all patients with PAH. Patients with PAH were classified into two groups: (i) those undergoing active treatment with phosphodiesterase 5 inhibitors, endothelin receptor antagonists, and/or intravenous prostacyclin; and (ii) “treatment-naïve” patients (i.e. those not undergoing any active treatment regimen). Finally, patients with diabetes mellitus, obesity, and coronary artery diseases were analyzed separately because the inflammatory responses associated with these conditions may affect PTX3 levels.

### Statistical Analysis

Plasma concentrations of PTX3, BNP, and CRP are given as the mean±SE. Statistical analyses were performed using SPSS version 17.0 (SPSS Inc., Chicago, IL, USA). Parametric tests, such as analysis of variance (ANOVA), were used after log transformation of the data, because PTX3 values did not exhibit normal distribution, but approximated a log-normal distribution [16]. Plasma concentrations of PTX3 were compared between the patient groups and healthy controls by Student’s *t*-test. Differences in plasma PTX3 concentrations between patients with and without active treatment, as well as between SSc patients with and without PAH, were evaluated the same manner. Two-tailed *P*<0.01 was considered significant. Pearson’s product–moment correlation coefficient was used to describe correlations between PTX3 and CRP or BNP after log transformation of the original data. Correlations between PTX3 and mPAP, PVR or disease duration were assessed by Spearman’s rank correlation coefficient. Receiver operating characteristic (ROC) curves were constructed to determine optimal threshold values for plasma PTX3. Areas under ROC curves (AUC_ROC_) and 95% confidence intervals (CI) were calculated to compare the effectiveness of PTX3 and BNP as markers of PAH.

## Results

### Patient Characteristics

In all, 184 subjects (50 PAH patients, 100 healthy controls, and 34 control CTD patients) were evaluated in the present study. In the group of PAH patients and healthy controls combined, there were 30 men and 120 women, with a mean (±SE) age at study entry of 52.6±1.2 years. As indicated in Table 1, the two groups were age and sex matched. Other patient characteristics are also given in Table 1. All PAH patients met the diagnostic criteria for PAH as specified in recent guidelines (1), and the presence of PAH was confirmed by right heart catheterization.

**Table 1:** Clinical characteristics of patients with pulmonary arterial hypertension and healthy controls.

	PAH patients(*n = *50)	Healthy controls(*n = *100)	*P*-value
Age (years)	51.0±2.4	53.3±1.4	0.377
No. women (%)	40 (80)	80 (80)	NS
No. with heart failure (%)	2 (4)	0	–
No. taking active treatment for PAH (%)	41 (82)	0	–
No. patients with diabetes mellitus (%)	0	0	NS
No. patients with obesity (%)	2	0	NS
No. patients with CAD (%)	0	0	NS
Pulmonary artery pressure (mmHg)	37.4±1.6	–	–
Pulmonary artery resistance (dyne.sec.cm^-5^)	691.0±64.6	–	–
Disease duration period (months)	33.3±4.4	–	–
Serum CRP (mg/dL)	0.14±0.04	–	–
Serum BNP (pg/mL)	113.2±28.6	–	–

Unless indicated otherwise, data are given as the mean ± SE.

PAH, pulmonary arterial hypertension; CAD, coronary artery diseases; CRP, C-reactive protein; BNP, B-type natriuretic peptide; NS, not significant.

### Plasma Concentrations of PTX3 and Other Biomarkers

**Figure 1:**
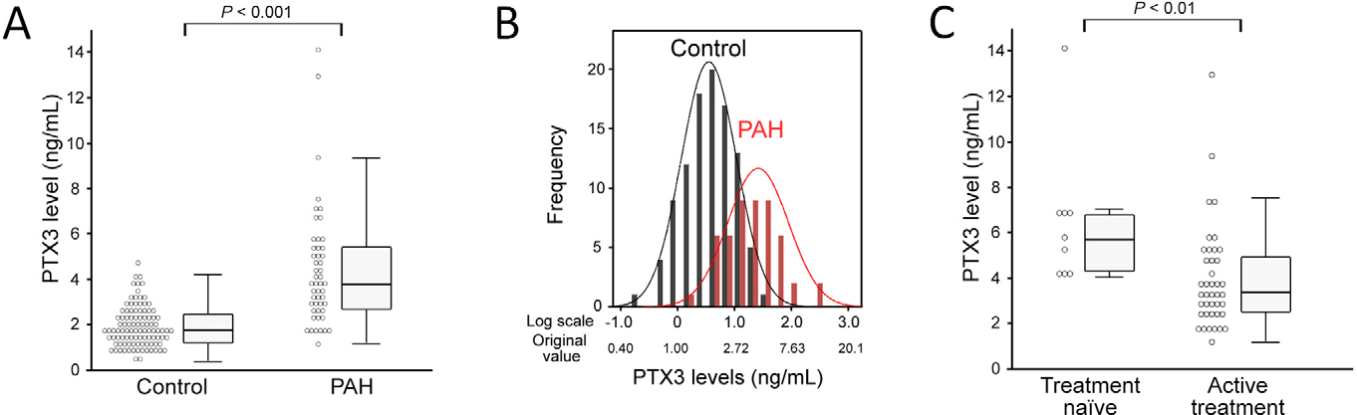
Serum pentraxin 3 (PTX3) concentrations in 50 patients with pulmonary arterial hypertension (PAH) and 100 healthy controls, and their correlation with serum concentrations of other biomarkers. A: Comparison of PTX3 concentrations in PAH patients and healthy controls. Mean plasma PTX3 concentrations were 4.40±0.37 and 1.94±0.09 ng/mL in the controls and PAH patients, respectively. B: Distribution of log-transformed PTX3 concentrations in PAH patients and healthy controls. C: Log-transformed PTX3 concentrations were significantly higher in patients with PAH than in healthy controls (1.34±0.07 vs. 0.55±0.05 log ng/mL, respectively; *P*<0.001). D, E: There was no correlation between plasma concentrations of PTX3 and either B-type natriuretic peptide (BNP; *r* = 0.33, *P* = 0.02) or C-reactive protein (CRP; *r* = 0.21, *P* = 0.14) in PAH patients.

**Figure 2:**
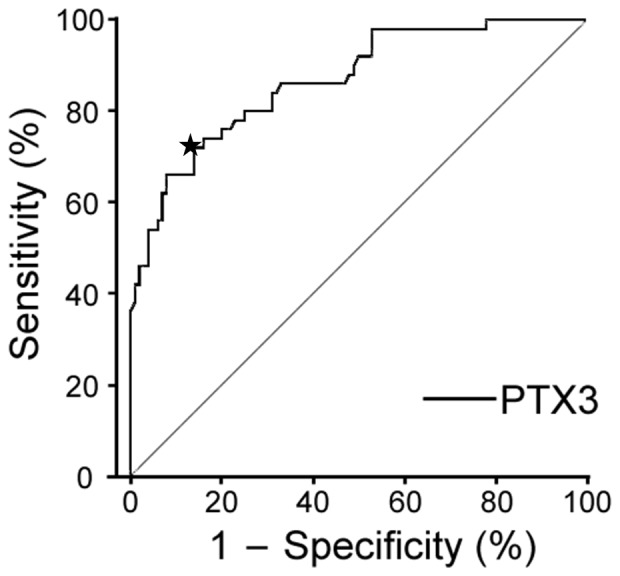
Receiver operating characteristic (ROC) curves for pentraxin 3 (PTX3). The area under the ROC curve was 0.866 (95% confidence interval 0.805–0.928). The star indicates the threshold concentration of 2.84 ng/mL PTX3 that maximized true-positive and false-negative results (sensitivity 74.0%, specificity 84.0%).

Mean plasma PTX3 concentrations in PAH patients were 4.40±0.37 ng/mL (range 1.18–14.11 ng/mL, median 3.83 ng/mL), compared with 1.94±0.09 ng/mL (range 0.39–4.60 ng/mL, median 1.78 ng/mL) in healthy subjects (Figure 1A). The log-transformed values of original plasma PTX3 concentrations approximated a symmetrical distribution in both healthy control group and patients with PAH group (Figure 1B). After log transformation, PTX3 concentrations in the PAH patients and healthy controls were 1.34±0.07 and 0.55±0.05 log ng/mL, respectively, revealing a significant increase in PTX3 concentrations in PAH patients compared with controls (*P*<0.001). In addition, BNP and CRP concentrations, hemodynamic parameters (mPAP and PVR), and disease duration were determined in patients with PAH. There were no significant correlations between PTX3 concentrations and either CRP (*r* = 0.21, *P* = 0.14) or BNP (*r* = 0.33, *P* = 0.02). Similarly, there were no significant correlations between PTX3 concentrations and mPAP (*r* = 0.13, *P* = 0.38), PVR (*r* = 0.15, *P* = 0.42), or disease duration (*r* = 0.17, *P* = 0.24). Conversely, significantly higher PTX3 concentrations were found in treatment-naïve patients (6.47±1.03 ng/mL, median 5.70 ng/mL) compared with patients undergoing active treatment (3.95±0.04 ng/mL, median 3.38 ng/mL; *P*<0.01; Figure 1C). The ROC curves indicated that PTX3 (AUC_ROC_ 0.866; 95% CI 0.805–0.928) is a potent biomarker for PAH (Figure 2). Using a threshold of 2.84 ng/mL, PTX3 maximized true-positive and false-negative results (sensitivity 74.0%, specificity 84.0%).

### Plasma PTX3 as a Screening Biomarker for PAH Associated with CTD

From the PAH patient cohort, 17 patients with CTD were chosen for comparison with 34 disease-matched control patients who had CTD (SSc or non-SSc) but not PAH. The two groups were matched for age, gender, and the type of CTD (see Table 2). As noted in the Methods section, PAH was ruled out on the basis of right heart catheterization and/or UCG results.

**Table 2:** Clinical characteristics and biomarkers in patients with connective tissue disease, with or without pulmonary arterial hypertension.

	CTD-PAH(*n* = 17)	CTD alone(*n* = 34)	*P*-value
Age (years)	56.3±4.6	56.3±2.7	0.990
No. women (%)	15 (88)	31 (91)	0.745
No. with SSc (%)	10 (59)	20 (59)	1
No. with heart failure (%)	1 (6)	0	–
No. being treated forPAH (%)	17 (100)	0	–
Serum PTX3 (mg/dL)	5.02±0.69	2.40±0.14	<0.001
Serum CRP (mg/dL)	0.24±0.09	0.22±0.04	0.936
Serum BNP (pg/mL)	189.3±74.4	49.3±12.1	0.014

Unless indicated otherwise, data are given as the mean ± SE.

CTD, connective tissue disease; PAH, pulmonary arterial hypertension; SSc, scleroderma; CRP, C-reactive protein; BNP, B-type natriuretic peptide; PTX3, pentraxin 3.

Mean plasma PTX3 concentrations in the CTD-PAH and CTD patients were 5.02±0.69 ng/mL (range 1.82–12.94 ng/mL) and 2.40±0.14 ng/mL (range 0.70–4.29 ng/mL), respectively (Table 2). Log transformation of the data revealed significantly higher PTX3 levels in CTD-PAH than in CTD patients (1.49±0.12 vs. 0.82±0.06 log ng/mL, respectively; *P*<0.001). Conversely, there were no significant differences in CRP levels between the two groups, and BNP levels in CTD-PAH patients were tend to higher than those in CTD patients but not significant (Table 2). In addition, we evaluated the correlation between PTX3 levels and levels of BNP and/or CRP in patients with CTD; however, we failed to find any significant correlations (*P* = 0.15 and 0.94, respectively; data not shown).

**Figure 3:**
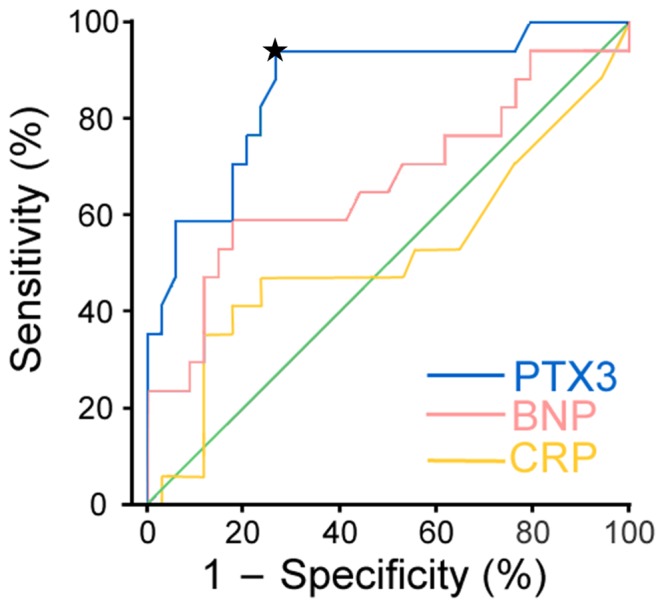
Receiver operating characteristic (ROC) curves for pentraxin 3 (PTX3) and other biomarkers in patients with connective tissue disease (CTD). The areas under the ROC curve (AUC_ROC_) for PTX3 was 0.866 (95% confidence interval (CI) 0.757–0.974). The star indicates the threshold concentration of 2.85 ng/mL PTX3 that maximized true-positive and false-negative results (sensitivity 94.1%, specificity 73.5%). The AUC_ROC_ for C-reactive protein (CRP) was 0.518 (95% CI 0.333–0.704), whereas that for B-type natriuretic peptide (BNP) was 0.670 (95% CI 0.497–0.842).

The ROC curves revealed that PTX3 (AUC_ROC_ 0.866; 95% CI 0.757–0.974) was a more accurate marker of the presence of PAH than either CRP (AUC_ROC_ 0.518; 95% CI 0.333–0.704) or BNP (AUC_ROC_ 0.670; 95% CI 0.497–0.842; Figure 3). A threshold concentration of 2.85 ng/mL PTX3 maximized true-positive and false-negative results (sensitivity 94.1%, specificity 73.5%).

## Discussion

The present study is the first report regarding the usefulness of PTX3, a local vascular inflammatory marker, as a screening tool for PAH. We found significantly higher levels of PTX3 in PAH patients compared with controls. There was no correlation between PTX3 levels and those of the classic systemic inflammatory marker CRP, which is of no value in screening for PAH. Moreover, in patients with CTD, comparisons of AUC_ROC_ revealed that PTX3 is a more sensitive biomarker for PAH than BNP. In addition, the AUC_ROC_ for PTX3 determined in the present study was superior to that reported previously for BNP [7].

Elevated PTX3 levels have been reported in many types of cardiovascular disease, including acute coronary syndrome [12], [13], congestive heart failure [18], and heart failure with normal ejection fraction [19]. In addition, recent reports have demonstrated the usefulness of PTX3 as a vascular inflammatory marker for distinguishing the activity of Takayasu aortitis [15], [16].

Local inflammatory activation in the pulmonary vasculature has already been shown to play an important role in the establishment of PAH [20], particularly PAH associated with CTD [21]. Recent studies have investigated whether PTX3 has a role in vascular disease and angiogenesis. PTX3 is produced at sites of vascular inflammation not only by smooth muscle and endothelial cells, but also by macrophages infiltrating the lesion [11], [22], [23], [24], [25], [26]. Interestingly, it has been reported that activated monocytes/macrophages contribute to the establishment of PAH under hypoxic conditions, as well as in patients with SSc [27], [28]. Moreover, some studies investigating gene expression in peripheral blood mononuclear cells from patients with SSc have reported upregulated *PTX3* gene expression in addition to that of VEGF and other inflammatory compounds [29], [30]. These findings provide strong support for our contention that PTX3 may be a potent biomarker for the detection of PAH, especially in patients with CTD.

In the present study, we investigated whether PTX3, the regulation of which is independent of that of the systemic inflammatory marker CRP, was a useful biomarker for diagnosing PAH. We found that PTX3 may be a more sensitive biomarker for PAH than BNP, which is, to date, the most established biomarker for PAH, especially in patients with CTD-PAH. Our findings suggest that PTX3 does not reflect the cardiac burden due to the pulmonary hypertension, but rather the activity of pulmonary vascular degeneration because PTX3 levels were significantly decreased after active treatment specifically for PAH.

In conclusion, we found that determining PTX3 concentrations may be more useful than BNP measurements for the detection of PAH, especially among patients with CTD. A limitation of the present study is that it is a single center and cross-sectional case control study and, as such, does not confirm the causal relationship between PTX3 and PAH. Further multicenter prospective studies to confirm the findings of the present study in a broader spectrum of patients with PAH and to evaluate the relationship between the disease activities of PAH and increases in PTX3 are needed before PTX3 can be used routinely as a screening biomarker. Furthermore, the role of PTX3 in lung tissue remains to be determined.
